# Ascending Aorta Size at Birth Predicts White Matter Microstructure in Adolescents Who Underwent Fontan Palliation

**DOI:** 10.1161/JAHA.118.010395

**Published:** 2018-12-14

**Authors:** Abbas H. Zaidi, Jane W. Newburger, David Wypij, Christian Stopp, Christopher G. Watson, Kevin G. Friedman, Michael J. Rivkin, Caitlin K. Rollins

**Affiliations:** ^1^ Department of Cardiology Boston Children's Hospital Boston MA; ^2^ Department of Neurology Boston Children's Hospital Boston MA; ^3^ Department of Psychiatry Boston Children's Hospital Boston MA; ^4^ Department of Radiology Boston Children's Hospital Boston MA; ^5^ Department of Pediatrics Harvard Medical School Boston MA; ^6^ Department of Neurology Harvard Medical School Boston MA; ^7^ Department of Biostatistics Harvard T.H. Chan School of Public Health Boston MA

**Keywords:** brain, congenital heart disease, echocardiography, magnetic resonance imaging, Congenital Heart Disease, Clinical Studies, Magnetic Resonance Imaging (MRI), Pediatrics

## Abstract

**Background:**

In neonates with single ventricle, smaller ascending aorta diameter is associated with cerebral white matter (WM) microstructural abnormalities. We sought to determine whether this association persists into adolescence.

**Methods and Results:**

Ascending aorta *Z* scores were obtained from first postnatal echocardiogram. Brain magnetic resonance imaging with diffusion tensor imaging was acquired in adolescence and used to obtain fractional anisotropy, axial diffusivity, radial diffusivity, and mean diffusivity in 33 WM tract regions of interest. Partial Pearson correlation coefficients were evaluated for associations between ascending aorta *Z* scores and WM microstructure measures, adjusting for sex, age at magnetic resonance imaging, scanner field strength, and Norwood status. Among 42 single ventricle patients aged 10 to 19 years, 31 had undergone the Norwood procedure as neonates. Lower ascending aorta *Z* scores were associated with lower fractional anisotropy in bilateral pontine crossing tracts (*P*=0.02), inferior fronto‐occipital fasciculus (*P*=0.02), and inferior longitudinal fasciculus (*P*=0.01); left cingulum–cingulate bundle (*P*=0.01), superior longitudinal fasciculus (*P*=0.04), and superior longitudinal fasciculus–temporal component (*P*=0.01); and right cingulum–hippocampal bundle *(P*=0.009) and inferior cerebellar peduncle (*P*=0.01). Lower ascending aorta *Z* scores were associated with higher radial diffusivity and mean diffusivity in a similar regional pattern but not with axial diffusivity.

**Conclusions:**

In adolescents with single ventricle, smaller aorta diameter at birth is associated with abnormalities of WM microstructure in a subset of WM tracts, mostly those located in deeper brain regions. Our findings suggest that despite multiple intervening medical or surgical procedures, prenatal cerebral blood flow may have a lasting influence on WM microstructure in single‐ventricle patients.

**Clinical Perspective**

**What Is New?**

- This study is the first to examine the relationship between aortic diameter at birth, a proxy for fetal cerebral blood flow, and adolescent cerebral white matter microstructure.
- Smaller aortic diameter at birth was associated with abnormal microstructure in a subset of white matter tracts.

**What Are the Clinical Implications?**

- Abnormal prenatal cerebral blood flow may have a long‐term influence on structural brain development.
- Further studies should clarify the relationship between prenatal cerebral blood flow and neurodevelopmental outcome.

## Introduction

Neurodevelopmental outcomes in children and adolescents with congenital heart disease (CHD) have shown only modest improvement over the past decade despite substantial changes in perioperative management.^1^, ^2^, ^3^ Recognizing that cardiac surgical management factors account for only a small percentage of the variance in outcomes, recent research efforts have explored how brain development is affected by patient factors, such as perinatal cerebral oxygen delivery and innate genetic abnormalities.^4^, ^5^, ^6^, ^7^

Infants with CHD, particularly those with single ventricle (SV), are at high risk of cerebral white matter (WM) injury. WM injury is present in 15% to 40% of infants undergoing neonatal surgery, emerging before or after surgery.^8^, ^9^ In a majority of cases, such injury no longer appears on routine neuroimaging within 3 months, although more sensitive quantitative magnetic resonance imaging (MRI) techniques can detect WM abnormalities years later.^8^, ^10^, ^11^, ^12^ Specifically, both SV and 2‐ventricle populations with CHD have been shown to have widespread reductions in WM volume and abnormal WM microstructure in late infancy through adolescence.^12^, ^13^, ^14^ Moreover, these abnormalities are associated with worse neurodevelopmental outcomes.^12^, ^14^

Reduced cerebral blood flow (CBF) in the fetal period may influence long‐term WM development. WM tracts begin forming as early as the first trimester of pregnancy, with additional tracts emerging and maturing throughout gestation. Transient cerebral compartments that appear during the fetal period—such as the intermediate and subplate zones—are critical for establishing the infrastructure for WM connections and may be specifically vulnerable to hypoxia–ischemia.^15^, ^16^, ^17^, ^18^ Indeed, neonates with CHD have dysmature WM microstructural development at birth.^19^ Smaller ascending aorta diameter at birth, an indicator of reduced prenatal CBF, has been linked to abnormal WM microstructure in SV neonates even before they undergo cardiac surgery.^20^

It is unknown whether the association in the neonate between abnormalities of WM microstructure and smaller ascending aorta diameter persists into later childhood and adolescence. We sought to determine whether reduced antegrade CBF prenatally correlates with long‐term alteration of WM microstructure despite the intervening medical and surgical events in an SV population. We hypothesized that smaller ascending aorta diameter at birth would predict altered WM microstructure in adolescence.

## Methods

The data, analytic methods, and study materials will be made available to other researchers for purposes of reproducing the results or replicating the procedure, upon request.

### Participants

Earlier publications have described recruitment details and data obtained in our cohort of Fontan children and adolescents who underwent brain MRI and neurodevelopmental evaluation.^21^, ^22^ Briefly, patients who had previously undergone Fontan palliation were recruited between 2010 and 2012 at Boston Children's Hospital. Eligible patients were 10 to 19 years of age at enrollment, had SV heart disease, and underwent testing at least 6 months after their open heart surgery. Patients were excluded if they had contraindications to MRI or if their primary language was not English. Parents and patients at least 18 years of age provided informed consent, and patients <18 years of age provided assent. For the current ancillary study, we analyzed the subset of patients who had available ascending aorta diameter and body surface area measurements from their first echocardiogram after birth, in addition to diffusion tensor imaging (DTI) in late childhood or adolescence. This study was approved by the Boston Children's Hospital institutional review board and adhered to institutional guidelines.

### Data Obtained

The parent Fontan study collected information on participant characteristics, surgical‐ and catheter‐based interventions, and complications.^22^ Participant characteristics explored in the current analysis included sex, race, ethnicity, genetic abnormality, birth weight, and gestational age at birth. Medical history variables included age at first operation; Norwood status; open first operation (versus shunt); deep hypothermic circulatory arrest (DHCA) and total support duration at first operation; complications at first operation; total numbers of operations, catheterizations, and their complications; and incidence of seizure, stroke, or any neurological event (ie, seizure, stroke, choreoathetosis, or meningitis).

Ascending aorta diameter and body surface area were extracted from a clinical database of the first echocardiogram after birth. From these data, we calculated ascending aorta *Z* scores (AoZ scores) as ascending aorta diameter adjusted for body surface area.^23^ The majority of patients with available ascending aorta dimensions at birth had undergone a Norwood operation.

Methods regarding DTI acquisition and analysis have been reported previously.^24^ Briefly, participants were scanned on either a 3‐ or 1.5‐T Twinspeed scanner (General Electric Medical Systems) including a diffusion weighted spin‐echo echo‐planar sequence: repetition/echo time=15 s/83.8 ms; flip angle=90°; acquisition matrix=96×96; field of view=240 mm, with resultant voxel size=2.5×2.5×2.5 mm^3^. Twenty‐five diffusion‐weighted gradient directions were acquired at b=1000 s/mm^2^ and 1 non–diffusion‐weighted image at b=0 s/mm^2^. DTI data were processed using the FMRIB Software Library v5.0.6 (University of Oxford). Raw diffusion data were corrected for movement and eddy current artifacts, then skull stripped.

Fractional anisotropy (FA), axial diffusivity, radial diffusivity (RD), and mean diffusivity (MD) were calculated by fitting a tensor model at every voxel, providing an indirect measure of WM microstructural organization through measurement of water mobility. FA is a weighted average of the eigenvalues of the diffusion tensor.^25^ Axial diffusivity is the principal eigenvalue of the diffusion tensor representing the principal diffusion direction of water. RD represents the amount of water diffusion perpendicular to the principal diffusion direction and has been associated with disruption of myelination or diminished axonal density. MD is a simple average of the eigenvalues.

We calculated the mean of each diffusion measure for each participant in 33 regions of interest determined by 2 atlases: the Johns Hopkins University WM tractography atlas and the DTI‐81 atlas.^26^, ^27^ These 2 atlases were selected to separately address hemispheric and brainstem/projection tracts, respectively. The regions of interest included the following tracts bilaterally: anterior thalamic radiation, cerebral peduncle, cingulum–cingulate bundle, cingulum–hippocampus bundle, corticospinal tract, external capsule, inferior cerebellar peduncle, inferior fronto‐occipital fasciculus, inferior longitudinal fasciculus, medial lemniscus, superior cerebellar peduncle, superior longitudinal fasciculus (SLF), SLF–temporal component, and uncinate fasciculus. Interhemispheric tracts were body of the corpus callosum, forceps major, forceps minor, middle cerebellar peduncle, and pontine crossing tract (PCT).

### Statistical Analysis

Group comparisons were examined by Fisher exact tests for categorical measures and 2‐sample *t* tests with equal variance or Wilcoxon rank sum tests for normal or nonnormal continuous distributions, respectively. Partial Spearman correlation coefficients and linear regression adjusting for Norwood status were used to examine the relationships of AoZ scores with participant and medical history characteristics. For closed procedures, values of DHCA and total support duration were set to 0. Descriptive statistics for the DTI measures were calculated with values of axial diffusivity, RD, and MD multiplied by 1000 for reporting purposes. Partial Pearson correlation coefficients adjusting for sex, age at MRI, scanner field strength, and Norwood status were used to examine relationships of AoZ scores with DTI measures. Given the exploratory nature of the analysis, we did not adjust for multiple comparisons. SAS 9.4 (SAS Institute) was used for all analyses, and all tests were 2‐sided.

## Results

### Participants

Of 144 Fontan participants who underwent MRI, 102 participants (71%) had available regional DTI measurements. Among those, AoZ scores were available for 42 participants, measured at a median of 0 (range: 0–42) days of age (Table S1). Among those Fontan participants with DTI measurements, participants with versus without available AoZ score data were younger at first operation and were more likely to be neonates, to have undergone a Norwood procedure, and to have a first operation that used cardiopulmonary bypass and DHCA. Those with available AoZ score data had longer DHCA duration, longer total support duration, and more operative complications at first operation; had more total open operations and total operative complications; were less likely to have had a stroke; and were younger at MRI. Participants with versus without AoZ score data were not significantly different with respect to sex, race, ethnicity, genetic abnormality, birth weight, gestational age at birth, total operations, total number of catheterizations and their complications, and incidence of seizure or any neurological event.

Table 1 describes the sociodemographic, echo, and medical history characteristics, stratified by Norwood status, of participants in the current study. Those who underwent a Norwood procedure (n=31), compared with participants who did not (n=11), had significantly lower AoZ scores (*P*<0.001); at first operation, were more likely to be a neonate (*P*=0.049), were more likely to have undergone an open procedure (*P*<0.001), and to have had more operative complications (*P*=0.04); and had more total open operations (*P*<0.001).

**Table 1:** Participant, Echocardiogram, and Medical History Characteristics of Fontan Participants With AoZ Scores and DTI Data

Variable	Norwood (n=31)	Non‐Norwood (n=11)	*P* Value*
Participant characteristics			
Male sex, n (%)	24 (77)	6 (55)	0.24
Race, n (%)			>0.99
Black	2 (6)	1 (9)	
White	29 (94)	10 (91)	
Hispanic ethnicity, n (%)	1 (3)	1 (9)	0.46
Genetic abnormality, n (%)	10 (32)	3 (27)	>0.99
Birth weight, kg, mean±SD	3.3±0.6	3.2±0.7	0.44
Gestational age, wk, mean±SD	39.0±2.2	38.3±2.1	0.32
Echocardiogram characteristics			
Age, d, median (range)	0 (0–42)	0 (0–35)	0.57
AoZ score, mean±SD	−2.5±1.5	0.2±1.1	<0.001
Echocardiogram to first operation, d, median (range)	3 (1–14)	2 (1–532)	0.78
Medical history			
Status at first operation			
Age, d, median (range)	4 (1–44)	7 (2–532)	0.35
Neonatal status (age ≤30 d), n (%)	30 (97)	8 (73)	0.049
Open procedure, n (%)	31 (100)	4 (36)	<0.001
Participants undergoing DHCA, n (%)	26 (90)	2 (50)	0.10
DHCA duration, min, median (range)	49 (0–107)	26.5 (0–93)	0.64
Total support duration, min, median (range)	127 (83–325)	111.5 (43–191)	0.19
Number of operative complications, median (range)	2 (0–10)	0 (0–3)	0.04
Total operations, median (range)	3 (2–4)	3 (1–4)	0.19
Total open operations, median (range)	3 (2–4)	2 (1–3)	<0.001
Total operative complications, median (range)	3 (0–12)	4 (0–6)	0.56
Total catheterizations, median (range)	4 (2–8)	4 (1–8)	0.21
Total catheterization complications, median (range)	1 (0–4)	1 (0–4)	0.71
Seizure, n (%)	6 (20)	0	0.17
Stroke, n (%)	1 (3)	0	>0.99
Any neurological event, n (%)†	7 (23)	0	0.16
Concurrent measures			
Age at MRI, y, mean±SD	14.3±2.9	12.6±2.4	0.10
Field strength, 3T, n (%)	16 (52)	5 (45)	>0.99
Family social status, mean±SD‡	46±14	49±12	0.62

AoZ score indicates ascending aorta *Z* score; DHCA, deep hypothermic cardiac arrest; DTI, diffusion tensor imaging; MRI, magnetic resonance imaging.

* *P* values for group comparisons were determined by Fisher exact tests for categorical measures, 2‐sample *t* tests with equal variance for continuous measures represented with means, and Wilcoxon rank sum tests for continuous measures represented with medians.

† Includes seizure, stroke, choreoathetosis, and meningitis.

‡ Score on Hollingshead Four‐Factor Index of Social Status, with higher scores indicating higher social status.

### Analysis of AoZ Scores With Participant and Medical History Characteristics

In regression analyses adjusting for Norwood status, AoZ scores were significantly lower in participants who underwent DHCA during their first operation compared with those who did not (β estimate: −2.3 [95% confidence interval, −3.8 to −0.8], *P*=0.004). Furthermore, lower AoZ scores were associated with longer DHCA duration at first operation (partial Spearman *r*=−0.35, *P*=0.03), longer total support duration at first operation (*r*=−0.34, *P*=0.04), and fewer total catheterization complications (*r*=0.38, *P*=0.01). AoZ scores were not significantly associated with participant characteristics, such as birth weight or gestational age at birth, or with other medical history variables, such as age at first operation, total operations, or total operative complications.

### Analysis of AoZ Scores With DTI Measures

Average DTI measures for regions of interest are provided in Table S2. Associations of AoZ scores with the DTI measures adjusting for sex, age at MRI, scanner field strength, and Norwood status are provided in Table 2. AoZ scores correlated with FA, with lower AoZ scores associated with lower FA in the PCT; bilaterally in the inferior fronto‐occipital fasciculus and inferior longitudinal fasciculus; in the left cingulum–cingulate bundle, SLF, and SLF–temporal component (Figure); and in the right cingulum–hippocampus bundle and inferior cerebellar peduncle. Lower AoZ scores were associated with higher RD bilaterally in the cingulum–hippocampus bundle and SLF, in the left cingulum–cingulate bundle and SLF–temporal component, and in the right external capsule and inferior cerebellar peduncle. Similarly, lower AoZ scores were associated with higher MD bilaterally in the SLF as well as in the left cingulum–hippocampus bundle and SLF–temporal component. No significant associations were observed between AoZ scores and axial diffusivity.

**Table 2:** Partial Pearson Correlation Coefficients of AoZ Scores With WM ROI Measures (n=42)

ROI	FA	AD	RD	MD
Body of the corpus callosum	0.20 (0.23)	−0.23 (0.16)	−0.25 (0.13)	−0.29 (0.08)
Forceps major	0.28 (0.08)	0.20 (0.22)	−0.20 (0.23)	−0.05 (0.75)
Forceps minor	0.17 (0.29)	−0.16 (0.34)	−0.23 (0.17)	−0.22 (0.18)
Middle cerebellar peduncle	0.23 (0.16)	0.04 (0.79)	−0.18 (0.28)	−0.09 (0.58)
PCT	0.37 (0.02)	0.08 (0.62)	−0.24 (0.14)	−0.15 (0.37)
Anterior thalamic radiation, LH	0.26 (0.12)	−0.12 (0.47)	−0.24 (0.14)	−0.23 (0.17)
Anterior thalamic radiation, RH	0.28 (0.09)	0.15 (0.38)	−0.17 (0.30)	−0.06 (0.72)
Cerebral peduncle, LH	0.25 (0.12)	0.16 (0.33)	−0.17 (0.31)	−0.05 (0.77)
Cerebral peduncle, RH	0.17 (0.30)	0.13 (0.43)	−0.10 (0.56)	0.01 (0.95)
Cingulum–cingulate bundle, LH	0.39 (0.01)	−0.03 (0.88)	−0.39 (0.02)	−0.30 (0.07)
Cingulum–cingulate bundle, RH	0.24 (0.15)	0.07 (0.67)	−0.14 (0.41)	−0.05 (0.77)
Cingulum–hippocampus bundle, LH	0.18 (0.27)	−0.15 (0.38)	−0.36 (0.03)	−0.33 (0.04)
Cingulum–hippocampus bundle, RH	0.42 (0.009)	0.17 (0.32)	−0.46 (0.003)	−0.23 (0.17)
Corticospinal tract, LH	0.11 (0.52)	−0.18 (0.27)	−0.21 (0.21)	−0.24 (0.14)
Corticospinal tract, RH	0.05 (0.76)	−0.22 (0.18)	−0.22 (0.19)	−0.29 (0.08)
External capsule, LH	0.25 (0.12)	0.04 (0.81)	−0.27 (0.11)	−0.17 (0.31)
External capsule, RH	0.30 (0.06)	0.03 (0.87)	−0.33 (0.04)	−0.23 (0.17)
Inferior cerebellar peduncle, LH	0.31 (0.05)	0.12 (0.46)	−0.19 (0.26)	−0.08 (0.62)
Inferior cerebellar peduncle, RH	0.40 (0.01)	−0.04 (0.81)	−0.33 (0.046)	−0.24 (0.15)
Inferior fronto‐occipital fasciculus, LH	0.36 (0.02)	0.18 (0.27)	−0.31 (0.06)	−0.15 (0.38)
Inferior fronto‐occipital fasciculus, RH	0.36 (0.03)	0.15 (0.35)	−0.24 (0.15)	−0.09 (0.59)
Inferior longitudinal fasciculus, LH	0.41 (0.01)	0.13 (0.42)	−0.30 (0.07)	−0.16 (0.35)
Inferior longitudinal fasciculus, RH	0.38 (0.02)	0.15 (0.36)	−0.26 (0.11)	−0.12 (0.47)
Medial lemniscus, LH	0.05 (0.75)	−0.09 (0.59)	−0.11 (0.51)	−0.12 (0.48)
Medial lemniscus, RH	0.19 (0.26)	0.09 (0.58)	−0.12 (0.46)	−0.04 (0.80)
Superior cerebellar peduncle, LH	0.04 (0.82)	0.01 (0.95)	−0.08 (0.62)	−0.05 (0.76)
Superior cerebellar peduncle, RH	0.26 (0.11)	−0.02 (0.90)	−0.27 (0.10)	−0.20 (0.23)
SLF, LH	0.34 (0.04)	−0.16 (0.35)	−0.41 (0.01)	−0.40 (0.01)
SLF, RH	0.23 (0.17)	−0.17 (0.29)	−0.37 (0.02)	−0.36 (0.03)
SLF–temporal component, LH	0.39 (0.01)	−0.17 (0.31)	−0.45 (0.004)	−0.46 (0.004)
SLF–temporal component, RH	0.17 (0.30)	−0.11 (0.50)	−0.28 (0.09)	−0.27 (0.10)
Uncinate fasciculus, LH	0.20 (0.22)	0.15 (0.37)	−0.15 (0.36)	−0.05 (0.78)
Uncinate fasciculus, RH	0.20 (0.22)	0.09 (0.61)	−0.15 (0.36)	−0.08 (0.63)

Values are partial Pearson *r* (*P* value). *P* values were determined by partial Pearson correlation coefficients adjusting for sex, age at magnetic resonance imaging, scanner field strength, and Norwood status. AD indicates axial diffusivity; AoZ score, ascending aorta *Z* score; FA, fractional anisotropy; LH, left hemisphere; MD, mean diffusivity; PCT, pontine crossing tract; RD, radial diffusivity; RH, right hemisphere; ROI, region of interest; SLF, superior longitudinal fasciculus; WM, white matter.

**Figure 1:**
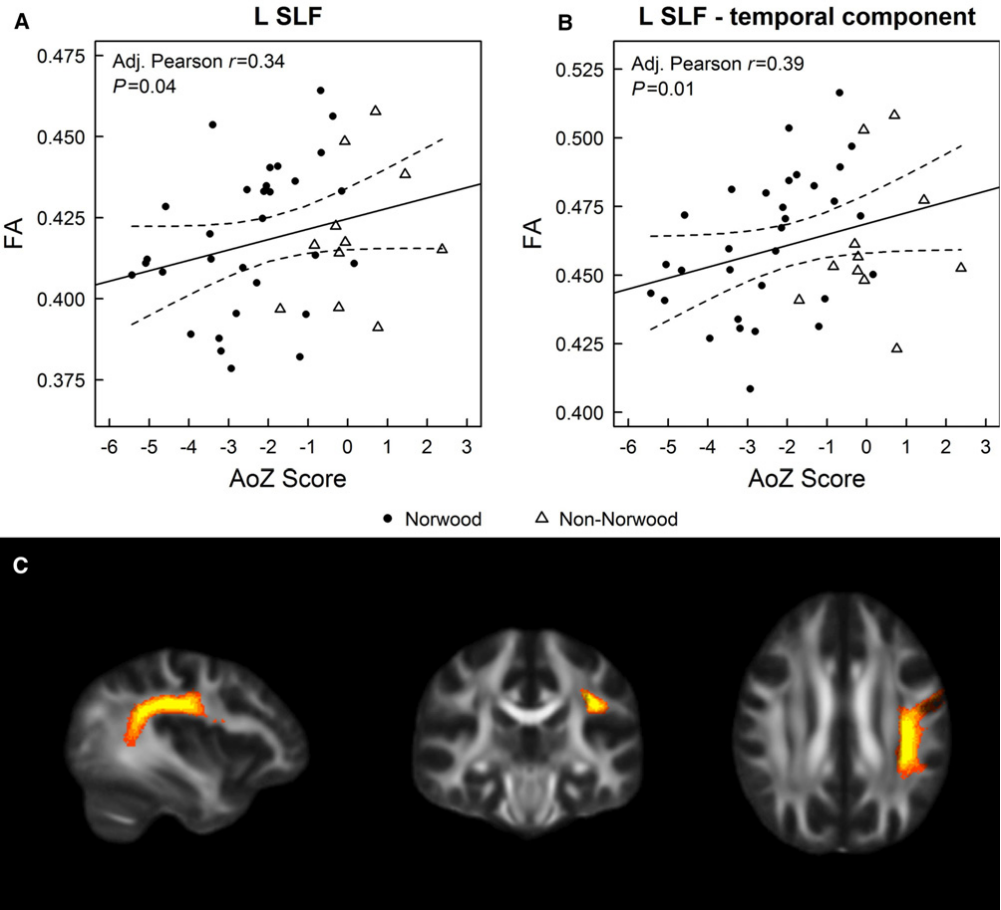
Relationships of ascending aorta *Z* scores (AoZ scores) with fractional anisotropy (FA) of the (**A**) left superior longitudinal fasciculus (L SLF) and (**B**) L SLF–temporal branch, stratified by Norwood status. **C**, L SLF in sagittal (left), coronal (middle), and axial (right) planes. “Hot” colors represent the voxel's probability of belonging to the L SLF, based on the Johns Hopkins University white matter tractography atlas.

## Discussion

We sought to determine whether prenatal CBF, using AoZ score at birth as its proxy, is associated with WM microstructure in adolescent patients with repaired SV. Our group previously identified widespread bihemispheric and brainstem abnormalities in WM microstructure among adolescents who underwent the Fontan procedure in early childhood.^24^ In this ancillary study of the same cohort, we found that smaller ascending aorta diameter at birth was associated with abnormalities of WM microstructure in a subset of WM tracts, especially those located in deeper regions of brain. Specifically, we found relationships between AoZ scores and WM microstructure in the PCT, inferior cerebellar peduncle, inferior fronto‐occipital fasciculus, inferior longitudinal fasciculus, and SLF regions and in tracts relating to the cingulum, cingulate, and hippocampus. In many of these tracts, lower AoZ scores were associated with lower FA and higher RD, which may reflect disruption of myelination or diminished axonal density. The region with the most consistent disruption of WM, as reflected by low FA and high RD and MD, was the left SLF, an important associative tract connecting the 4 lobes of the brain with a role in many cognitive functions, including attention, executive functions, and language.^28^, ^29^, ^30^ Overall, our findings suggest that despite the multiple intervening medical and surgical procedures, prenatal CBF may exert a lasting influence on WM microstructure in certain regions of brain.

Our findings are consistent with a growing body of evidence suggesting that reduced prenatal CBF and oxygen/nutrient delivery have a significant impact on brain development in CHD. Among a heterogeneous group of fetuses with CHD, those fetuses with a lower percentage of combined ventricular output from the aorta showed smaller total brain volumes.^31^ In the same study, the N‐acetyl aspartate:choline ratio, an indicator of neuronal and axonal health, was found to be lowest in those lacking antegrade blood flow through the aortic arch. Among those fetuses with hypoplastic left heart syndrome, who would be expected to have substantially reduced cerebral oxygenation and perfusion, regional brain volumes in cortical and subcortical gray matter and WM were smaller than in controls, and cortical development appeared delayed.^32^ Sun and colleagues measured cerebral oxygen consumption and found a positive relationship with total brain volume in a cohort of near‐term fetuses with heterogeneous CHD, and measures of oxygen delivery showed a trend in the same direction.^4^ Whereas prenatal CBF, using neonatal aorta diameter as a proxy, has been previously associated with WM microstructure at term, our cohort demonstrates that this association persists to adolescence in at least some regions of the brain.^20^ This relationship is particularly notable in our population, as SV patients with CHD undergo multiple subsequent medical and surgical stresses with potential for ongoing cerebral insult that might be expected to dominate prenatal factors.^33^, ^34^ A pattern of reduced FA with increased RD has been observed previously in WM of adolescents born preterm and very low birth weight.^33^, ^35^ It is possible that our finding of FA reduction and RD enhancement in adolescence and the correlation with reduced neonatal aorta diameter could reflect lasting WM injury sustained very early in life, including the prenatal period.

The mechanism through which reduced prenatal CBF and oxygen delivery may lead to abnormal WM development in these regions is uncertain. It is possible that the location of certain tracts may make them particularly vulnerable to reduced prenatal CBF in SV. Cerebral vasculature undergoes substantial development throughout the third trimester, with perfusion gradually increasing to deeper regions via branching of penetrating arteries.^36^ Areas with less mature vascular structure are more vulnerable to reduced cerebral perfusion, specifically WM in deep periventricular end zones as well as more diffuse subcortical end/border zones.^36^, ^37^ Furthermore, CBF has a gradient throughout the brain, with much lower flow rates in deep WM than in the cerebral cortex.^36^ Deep WM may be particularly vulnerable in fetuses with SV, in whom reduced cerebral perfusion may injure nascent oligodendrocytes only to compromise subsequent myelination, with lasting effects.^35^, ^38^ Many of the tracts where we found that WM microstructure correlated with aorta diameter at birth are located in deep regions that would be dependent on a less‐developed vasculature in the third trimester and potentially would be more vulnerable to low cerebral perfusion.

An alternative hypothesis is that the timing of emergence and maturation of different WM tracts creates a heightened vulnerability in certain developing tracts during the late fetal period.^39^ WM tracts in the brain emerge and develop at variable times; broadly speaking, limbic and commissural tracts tend to develop earliest, followed by projection and association tracts.^40^, ^41^ Except for the PCT, many of the regions in which we found correlations are association tracts that emerge by about 19 weeks of gestation and undergo significant maturation in the third trimester. Although all of the tracts examined could be adversely affected by deficient CBF that results in damage to nascent oligodendrocytes, the early‐developing tracts in which we identified associations would be especially good candidates for CBF‐related oligodendrocyte injury to result in abnormal myelination. This pattern would not fully explain our results; for example, the PCT undergoes substantial transformation before that time period. Nonetheless, rapid growth of several of these WM tracts in the late second and third trimesters may in part explain the pattern of associations we found between aorta diameter at birth and WM microstructure.

Our study has limitations. Smaller size of the aorta at birth is associated with greater operative complexity, including longer duration of DHCA and total support, which may confound our results. Of note however, in our prior DTI analysis with a larger sample, these medical variables were not independently associated with DTI measures. We did not have sufficient power to analyze the effect of aortic atresia on WM microstructure; however, we would anticipate that the patients with aortic atresia would have the smallest ascending aortas. Although we adjusted for Norwood status in our analyses, our sample size had insufficient power to detect effect modification by Norwood status. We did not find a widespread correlation between AoZ scores and WM microstructure. Aside from the PCT, the WM tracts found to be significantly associated with AoZ scores at term are a subset of those that were previously found to differ in post‐Fontan adolescents compared with a control group.^24^ We hypothesized that only certain tracts are affected by diminished prenatal CBF because of their location in brain or the timing of maturation; however, it is also possible that the lack of more widespread correlation is due to the small sample size in our study. Finally, given our small sample size, we also did not examine relationships to neurodevelopmental outcome measures. Our findings should be considered exploratory and interpreted with caution.

In conclusion, these data suggest that fetal CBF, using ascending aorta diameter at birth as its proxy, may have a lasting impact on cerebral WM microstructure in patients with CHD. Studies with a larger sample size are needed to determine the broader relationship among aorta diameter at birth, postnatal medical and surgical factors, and WM microstructure in adolescence.

## Sources of Funding

Research reported in this publication was supported by the National Heart, Lung, and Blood Institute of the National Institutes of Health (NIH) under award number R01 HL096825, the National Institute of Neurological Disorders and Stroke of the NIH under award number K23NS101120, an American Academy of Neurology Clinical Research Training Fellowship, and the Farb Family Foundation. The content is solely the responsibility of the authors and does not necessarily represent the official views of the NIH.

## Disclosures

None.

## Supporting information


**Table S1.** Participant, Echocardiogram, and Medical History Characteristics of Fontan Participants With Diffusion‐Tensor Imaging Data
**Table S2.** White Matter Region of Interest Measures (n=42)
