# Supplementary material for: Ascending Aorta Size at Birth Predicts White Matter Microstructure in Adolescents Who Underwent Fontan Palliation
Source: J Am Heart Assoc. 2018 Dec 14;7(24):e010395. doi: 10.1161/JAHA.118.010395 (PMC6405606; doi:10.1161/JAHA.118.010395)
Supplement: Supplementary file 1 — Table S1. Participant, Echocardiogram, and Medical History Characteristics of Fontan Participants With Diffusion‐Tensor Imaging Data Table S2. White Matter Region of Interest Measures (n=42) [file JAH3-7-e010395-s001.pdf]

SUPPLEMENTAL MATERIAL

Table S1. Subject, echocardiogram, and medical history characteristics of Fontan subjects with DTI data.

Variables	With AoZ score (n = 42)	Without AoZ score (n = 60)	*P* value*
Subject characteristics			
Male sex, n (%)	30 (71)	31 (52)	0.06
Race, n (%)			0.31
Asian	0	3 (5)	
Black	3 (7)	2 (3)	
Caucasian	39 (93)	55 (92)	
Hispanic ethnicity, n (%)	2 (5)	9 (15)	0.12
Genetic abnormality, n (%)	13 (31)	27 (45)	0.22
Birth weight, kg, mean ± SD	3.3 ± 0.6	3.2 ± 0.6	0.68
Gestational age, wk, mean ± SD	38.8 ± 2.1	38.9 ± 2.5	0.87
Echocardiogram characteristics			
Age, d, median (range)	0 (0 to 42)		
AoZ score, mean ± SD	–1.8 ± 1.8		
Echocardiogram to first operation, d, median (range)	3 (1 to 532)		
Medical history			
Status at first operation			

Age, d, median (range)	4.5 (1 to 532)	7.5 (1 to 757)	0.02
Neonatal status (age ≤ 30 d), n (%)	38 (90)	41 (68)	0.009
Norwood status, n (%)	31 (74)	9 (15)	<0.001
Open procedure, n (%)	35 (83)	22 (37)	<0.001
Subjects undergoing DHCA, n (%)	28 (85)	8 (47)	0.008
DHCA duration, min, median (range)	49 (0 to 107)	0 (0 to 66)	0.01
Total support duration, min, median (range)	125 (43 to 325)	86 (45 to 156)	<0.001
Number of operative complications, median (range)	2 (0 to 10)	0 (0 to 5)	<0.001
Total operations, median (range)	3 (1 to 4)	3 (1 to 5)	0.69
Total open operations, median (range)	3 (1 to 4)	2 (1 to 5)	<0.001
Total operative complications, median (range)	3 (0 to 12)	2 (0 to 9)	0.005
Total catheterizations, median (range)	4 (1 to 8)	4 (2 to 17)	0.35
Total catheterization complications, median (range)	1 (0 to 4)	0 (0 to 5)	0.10
Seizure, n (%)	6 (15)	6 (10)	0.54
Stroke, n (%)	1 (2)	9 (15)	0.04
Any neurological event, n (%)†	7 (17)	16 (27)	0.33
Concurrent measures			
Age at MRI, yr, mean ± SD	13.8 ± 2.9	15.3 ± 2.9	0.01

Field strength, 3T, n (%)	21 (50)	21 (35)	0.15

DTI indicates diffusion tensor imaging; AoZ, ascending aorta Z; DHCA, deep hypothermic cardiac arrest.

**P* values for group comparisons were determined by Fisher's exact tests for categorical measures, 2-sample *t* tests with equal variance for continuous measures represented with means, and Wilcoxon rank sum tests for continuous measures represented with medians.

†Includes seizure, stroke, choreoathetosis, and meningitis.

Table S2. WM ROI measures (n = 42).

ROIs	FA	AD (x1000)	RD (x1000)	MD (x1000)
Body of the corpus callosum	0.59 ± 0.05	1.59 ± 0.08	0.55 ± 0.07	0.89 ± 0.06
Forceps major	0.63 ± 0.03	1.45 ± 0.05	0.45 ± 0.04	0.79 ± 0.03
Forceps minor	0.44 ± 0.02	1.24 ± 0.06	0.55 ± 0.04	0.78 ± 0.04
Middle cerebellar peduncle	0.49 ± 0.02	1.10 ± 0.04	0.49 ± 0.02	0.69 ± 0.02
PCT	0.45 ± 0.03	1.08 ± 0.04	0.55 ± 0.04	0.72 ± 0.04
Anterior thalamic radiation, LH	0.40 ± 0.02	1.14 ± 0.04	0.61 ± 0.03	0.78 ± 0.03
Anterior thalamic radiation, RH	0.39 ± 0.02	1.14 ± 0.04	0.61 ± 0.02	0.78 ± 0.03
Cerebral peduncle, LH	0.61 ± 0.03	1.47 ± 0.06	0.49 ± 0.03	0.81 ± 0.03
Cerebral peduncle, RH	0.61 ± 0.02	1.47 ± 0.06	0.48 ± 0.02	0.81 ± 0.03
Cingulum-cingulate bundle, LH	0.46 ± 0.04	1.18 ± 0.06	0.55 ± 0.04	0.76 ± 0.04
Cingulum-cingulate bundle, RH	0.41 ± 0.04	1.13 ± 0.07	0.59 ± 0.05	0.77 ± 0.05
Cingulum-hippocampus bundle, LH	0.41 ± 0.04	1.20 ± 0.07	0.61 ± 0.04	0.81 ± 0.04
Cingulum-hippocampus bundle, RH	0.41 ± 0.04	1.17 ± 0.07	0.60 ± 0.04	0.79 ± 0.04
Corticospinal tract, LH	0.58 ± 0.02	1.32 ± 0.04	0.48 ± 0.02	0.76 ± 0.02
Corticospinal tract, RH	0.58 ± 0.02	1.32 ± 0.04	0.47 ± 0.02	0.76 ± 0.02
External capsule, LH	0.35 ± 0.02	1.13 ± 0.04	0.65 ± 0.03	0.81 ± 0.02

External capsule, RH	0.36 ± 0.02	1.14 ± 0.04	0.65 ± 0.03	0.81 ± 0.03
Inferior cerebellar peduncle, LH	0.45 ± 0.03	1.22 ± 0.05	0.59 ± 0.04	0.80 ± 0.03
Inferior cerebellar peduncle, RH	0.45 ± 0.03	1.18 ± 0.05	0.57 ± 0.04	0.78 ± 0.04
Inferior fronto-occipital fasciculus, LH	0.46 ± 0.02	1.23 ± 0.04	0.58 ± 0.03	0.80 ± 0.02
Inferior fronto-occipital fasciculus, RH	0.45 ± 0.02	1.22 ± 0.04	0.57 ± 0.03	0.79 ± 0.03
Inferior longitudinal fasciculus, LH	0.44 ± 0.02	1.24 ± 0.04	0.60 ± 0.03	0.81 ± 0.03
Inferior longitudinal fasciculus, RH	0.46 ± 0.02	1.25 ± 0.04	0.58 ± 0.03	0.80 ± 0.03
Medial lemniscus, LH	0.51 ± 0.03	1.32 ± 0.07	0.56 ± 0.04	0.81 ± 0.04
Medial lemniscus, RH	0.51 ± 0.03	1.30 ± 0.06	0.55 ± 0.04	0.80 ± 0.04
Superior cerebellar peduncle, LH	0.50 ± 0.03	1.46 ± 0.08	0.64 ± 0.05	0.91 ± 0.04
Superior cerebellar peduncle, RH	0.49 ± 0.03	1.46 ± 0.08	0.67 ± 0.05	0.93 ± 0.05
SLF, LH	0.42 ± 0.02	1.09 ± 0.03	0.56 ± 0.03	0.74 ± 0.02
SLF, RH	0.43 ± 0.02	1.10 ± 0.04	0.55 ± 0.03	0.73 ± 0.03
SLF - temporal component, LH	0.46 ± 0.03	1.14 ± 0.03	0.55 ± 0.03	0.74 ± 0.02
SLF - temporal component, RH	0.47 ± 0.03	1.17 ± 0.05	0.55 ± 0.03	0.75 ± 0.03
Uncinate fasciculus, LH	0.38 ± 0.03	1.13 ± 0.04	0.60 ± 0.03	0.78 ± 0.03
Uncinate fasciculus, RH	0.38 ± 0.03	1.11 ± 0.05	0.55 ± 0.03	0.74 ± 0.03

WM indicates white matter; ROI, region of interest; FA, fractional anisotropy; AD, axial diffusivity; RD, radial diffusivity; MD, mean diffusivity; PCT, pontine crossing tract; LH, left hemisphere; RH, right hemisphere; SLF, superior longitudinal fasciculus.

Values are means ± SD
